# Substrate profiling of marine-derived thermotolerant cysteine protease reveals unique cleavage preferences for industrial applications

**DOI:** 10.1038/s41598-025-11635-1

**Published:** 2025-07-21

**Authors:** Victoria Røyseth, Brianna M. Hurysz, Hasan Arsın, Julia M. Vazquez, Anna-Karina Kaczorowska, Anita-Elin Fedøy, Daria Biernacka, Sebastian Dorawa, Tadeusz Kaczorowski, Runar Stokke, Anthony J. O’Donoghue, Ida Helene Steen

**Affiliations:** 1 Department of Biological Sciences, Center for Deep Sea Research, University of Bergen, Bergen, Norway; 2 Skaggs School of Pharmacy and Pharmaceutical Sciences, University of California, San Diego, USA; 3 Collection of Plasmids and Microorganisms | KPD, Faculty of Biology, University of Gdańsk, Gdańsk, Poland; 4 Laboratory of Extremophiles Biology, Department of Microbiology, Faculty of Biology, University of Gdańsk, Gdańsk, Poland

**Keywords:** Marine cysteine protease, Globupain, Clostripain, Multiplex substrate profiling by mass spectrometry, Marine bioprospecting, Hydrothermal vents, Hydrolases, Enzymes, Proteases

## Abstract

Due to their industrial importance, new proteases are constantly being sourced from the marine environment. However, their substrate specificities remain insufficiently studied, restricting the evaluation of their potential applications. Here, we applied multiplex substrate profiling by mass spectrometry (MSP-MS) to globupain, a marine thermotolerant clostripain-like protease and show that it has a novel substrate specificity. Globupain is an endopeptidase with a preference for cleavage of substrates on the C-terminal side of norleucine (Nle), Leu, Asn, Arg and Lys. While it can hydrolyze gelatin and collagen, its reaction rate is lower than that of papain, a commercial cysteine protease. The precise knowledge of substrate specificity of globupain led to the discovery that the calpain inhibitors MG101 and leupeptin inactivate globupain activity with IC_50_ values of 23.79 and 138.7 nM, respectively. Further investigation of additive effects revealed that globupain activity was stimulated by Triton X-100 and Tween 40 at concentrations of up to 1%. Globupain exhibited tolerance to elevated DTT concentrations and retained most of its activity in the presence of Mg^2+^ or Mn^2+^ compared to its preferred cation, Ca^2+^. In conclusion, globupain is a novel clostripain-like cysteine protease with a distinct substrate cleavage profile and remarkable stability in the presence of various additives, highlighting its potential for industrial applications.

## Introduction

Proteases are enzymes that cleave peptide bonds in proteins and exhibit a diverse range of catalytic mechanisms and substrate preferences^1^. They function either as endopeptidases or exopeptidases and may possess narrow specificity, targeting only specific cleavage sites, or broad specificity, acting on a wider range of substrates. The substrate specificity of a protease provides valuable insight into its biological function^2^ and remains a key factor in determining its potential applications. Today, proteases account for a large fraction of commercial enzymes sold and used worldwide^3–5^, highlighting their economic importance. They have widespread applications across various industries, including pharmaceuticals, food production, detergents, and waste management^6,7^. The unique substrate specificity of a protease also influences the composition of the degradation end-products^8–10^. In some applications, such as proteomic studies, restricted specificity is advantageous. For example, the strict specificity of trypsin for Arg and Lys or of clostripain for only Arg at the P1 position^11^ is well recognized. In contrast, applications in commercial food processing and livestock feed often benefit from proteases with broad substrate specificity or combinations of different proteases to achieve the desired hydrolysate composition^10,12^. Understanding the unique cleavage preferences of proteases allows for their targeted use in specific processes, driving the development of novel applications and innovative products, thereby expanding market opportunities. Additionally, assessing how various factors, such as temperature, pH, salt, and detergent, affect proteolytic activity is crucial when evaluating enzymes for industrial applications^7,10,13–15^.

Driven by the need to uncover the functional roles of proteases in diverse biological processes, powerful and sensitive methods are constantly being developed to provide unique insights into their substrate specificities. Multiplex substrate profiling by mass spectrometry (MSP-MS) is a method that enables the detection and quantification of cleavage products from a library of synthetic peptides with diverse sequences. Cleavage sites are identified using liquid chromatography-tandem mass spectrometry (LC–MS/MS) by analyzing cleavage products and comparing them to the parent peptide. By examining the prime and non-prime amino acids surrounding each cleavage site, MSP-MS generates a comprehensive substrate specificity profile for the target protease. This analysis can be performed at any pH and temperature and in the presence of most additives commonly found in biological reaction buffers^16,17^. Previously, MSP-MS has been successfully applied to determine the substrate specificity of the C11 cysteine protease PmC11 from the human gut bacterium *Parabacteroides merdae*^18^ and the commercial C1 cysteine protease, papain^19^.

Recently, we reported the discovery and characterization of globupain, a thermoactive and thermotolerant clostripain-like C11 cysteine protease^20^. Globupain originates from an undescribed *Archaeoglobales* metagenome-assembled genome reconstructed from an Arctic hydrothermal vent system^20^. Comparative amino-acid sequence analysis revealed that globupain shares the highest identity with human gut and intestinal C11 proteases, including the PmC11 cysteine protease. When assessed using a library of 17 fluorogenic substrates, globupain exhibited a strong preference for Arg over Lys at the P1 position.

Here, we analysed globupain using MSP-MS in order to better understand its substrate preference. The determined substrate specificity of globupain guided the design of new fluorogenic substrates and facilitated the identification of potent inhibitors. Our approach highlights the benefits of utilizing MSP-MS to identify key enzymatic characteristics of novel proteases that have the potential for use in industrial applications.

## Methods

### Enzyme preparation and storage

Recombinant globupain was purified as previously described^20^ and stored at 4 °C in 20 mM trisodium citrate buffer pH 5.5 supplemented with 150 mM NaCl. The enzyme was activated by supplementing the reaction mixture with 1 mM CaCl_2_ and 2.5 mM DTT, followed by incubation at 75 °C for 4.5 h before rapid cooling on ice. The activated enzyme remains stable at 4 °C for up to six months without any loss of activity.

### Comparison of globupain activity to commercial cysteine proteases clostripain and papain

Globupain activity against 50 μM Boc-QAR-amc (Sigma-Aldrich) was evaluated by comparing with commercial cysteine proteases Clostripain (from *Clostridium histolyticum*, cat. no. C0888, Merck) and Papain (from *Carica papaya*, cat. no. 76216, Merck). To standardize the assay conditions across all enzymes, a reaction buffer (Assay Buffer 1) was used, comprising 20 mM citrate–phosphate buffer (pH 7.0), 150 mM NaCl, 1 mM CaCl₂, 10 mM DTT, and 0.5% (w/v) L-cysteine. Additionally, globupain was tested using Assay Buffer 2, which consisted of 20 mM citrate–phosphate buffer (pH 7.1), 150 mM NaCl, 1 mM CaCl₂, and 10 mM DTT, as previously described^20^. Each enzyme was assayed in triplicate at 60 °C with a final concentration of 1.45 µg/mL. Fluorescence measurements were taken every 20 s for 160 cycles using an EnSpire™ 2300 Multilabel Reader (PerkinElmer, Turku, Finland), with excitation at 360 nm and emission at 460 nm. Background fluorescence was determined for samples containing only the substrate and assay buffer. Reaction rates, expressed in relative fluorescence units per second (RFU/s), were calculated from the linear portion of the fluorescence curves using GraphPad Prism software (version 10.4.1).

### Globupain activity on gelatin and collagen, with comparison to commercial papain

To assess the activity of globupain against gelatin and collagen, the EnzChek™ Gelatinase/Collagenase Assay Kit (Invitrogen, Corp. Carlsbad, CA, USA) was used. The substrates employed were fluorescein-conjugates DQ™ collagen type I from bovine skin and DQ™ gelatin from pig skin (Thermo Fisher Scientific, MA, USA). The assay was performed according to the manufacturer’s protocol, using Assay Buffer 1 at 60 °C. Papain from *Carica papaya* (cat. no. 76216, Merck Millipore, Darmstadt, Germany) served as a comparative cysteine protease. Reaction mixtures were prepared by combining 40 µL of Assay Buffer 1 with 10 µL of 1 mg/mL substrate solution and 50 μL of an 11.6 µg/mL activated globupain or papain in a total reaction volume of 100 µL. The final amount of enzyme was 5.8 µg/mL. Samples were run in triplicate, and the background fluorescence was measured for samples containing only the substrate and assay buffer. Fluorescence measurements and data analysis were carried out as described above.

### Activity of globupain with increasing DTT, NaCl and CaCl₂ concentrations

Globupain activity was evaluated in Assay Buffer 2 with varying concentrations of DTT (0–20 mM), NaCl (0–1, 250 mM), or CaCl_2_ (0–25 mM). Reactions were carried out at 50 °C for 1 h using 50 μM Boc-QAR as the substrate and an enzyme concentration of 0.26 μg/mL. All assays were run in triplicate wells on a black 384-well plate (Thermo Fisher Scientific, MA, USA). Fluorescence was measured at an excitation of 360 nm and emission of 460 nm using a BioTek Synergy HTX Multimode Reader (BioTek, Agilent, TX, USA).

### Effect of divalent cations on globupain activity

The effect of divalent cations on globupain activity was investigated in the presence of 1 mM Mg^2+^ (MgSO₄), Mn^2+^ (MnCl₂), Cu^2+^ (CuSO₄), Fe^2+^ (FeSO₄), Zn^2+^ (ZnCl₂), and Co^2+^ (CoCl₂), with activity normalized to that observed with 1 mM Ca^2+^ (CaCl₂). Reactions were carried out in 20 mM citrate–phosphate buffer (pH 7.1), 10 mM DTT, 150 mM NaCl, 25 μM Boc-QAR-amc supplemented with 1 mM of each divalent cation, and 2.9 μg/mL of enzyme. Fluorescence resulting from Boc-QAR-amc degradation was measured at 50 °C using an EnSpire™ 2300 Multilabel Reader (PerkinElmer, Turku, Finland), following the previously reported protocol^20^. All reactions were performed in triplicate, and the data were analyzed using GraphPad Prism (version 10.4.1) software and RStudio 2024.09.1 Build 394 (RStudio, 2024).

### Effect of detergents on globupain activity

The effect of detergents and the chaotropic agent urea on globupain activity was tested at 75 °C. The enzyme, at a concentration of 5.9 μg/mL, was incubated in Assay Buffer 2 with 1% of each of the following compounds: CHAPS (Merck), SDS (Sigma), Triton X-100 (Sigma-Aldrich), Tween 20 (Sigma-Aldrich), Tween 40 (Sigma-Aldrich), Tween 80 (Sigma-Aldrich) and urea (Merck). For each reaction set, a control sample without detergent or urea was included. An aliquot was taken immediately after the enzyme was added to the reaction (0 min timepoint) and placed on ice. Subsequent aliquots were collected at 60 and 120 min and also placed on ice. At each time point, enzyme activity was assessed at 50 °C by mixing 50 µL of the collected aliquot with 50 µL of Assay Buffer 2 containing 100 µM Boc-QAR-amc. This experimental set-up ensured a final concentration of 0.5% of each additive. Enzyme activity was measured from triplicate samples at each time point. Background fluorescence was measured for samples containing only the reaction buffer, substrate and detergent or urea at a final concentration of 0.5%.

### Effect of detergents on globupain stability

The conformational stability of activated globupain with 1% addition in respective detergents was analyzed by thermal denaturation using nano-differential scanning fluorimetry (nanoDSF). Activated globupain (0.3 mg/mL) in 150 mM citrate phosphate buffer (pH 7.1), 10 mM DTT, and 1 mM CaCl_2_ was equilibrated to room temperature and then briefly centrifuged (10,000×*g*, 1 min) to remove residual air bubbles and pre-existing protein aggregates. Standard-grade glass capillaries were filled with 10 μL of the sample, sealed with capillary sealing paste, and placed in a Prometheus NT.48 instrument (NanoTemper Technologies, Munich, Germany), which measures intrinsic protein fluorescence at 330 and 350 nm over a temperature gradient. All measurements were conducted using a temperature ramp from 20 to 110 °C at a heating rate of 1 °C/min. The acquisition was followed by data analysis using the PR.ThermControl v.2.3.1 software (NanoTemper Technologies). The melting temperature (T_m_), defined as the temperature at which half the protein molecules are unfolded, was determined using the inflection point (IP350/330) of the first derivative. In nano DSF experiments, Triton X-100 reduced (cat. no. X100RS; Sigma-Aldrich) was used.

### Multiplex substrate profiling by mass spectrometry (MSP-MS)

A defined library of 228 14-mer peptides, containing 2,964 unique potential cleavage sites, was used as a substrate pool to investigate the substrate specificity of globupain^16,17^. Purified and activated globupain^20^ was mixed with an equimolar mixture of all 228 peptides in assay buffer (100 mM citrate phosphate, 10 mM DTT, 1 mM CaCl₂, and 150 mM NaCl at pH 7.1) such that the final concentration of enzyme was 0.588 µg/mL, and each peptide was 0.5 μM. Samples were prepared in quadruplicate reactions, incubated at 75 °C, and inactivated by mixing 1:5 with 8 M urea at time intervals of 15 min, 1 h, and 4 h. Quadruplicate negative control samples were prepared by immediately inactivating them with 8 M urea. Subsequently, after inactivation, samples were desalted, dried down, and rehydrated in 0.1% trifluoroacetic acid. Each sample was subjected to mass spectrometry analysis, and the raw data files were processed using PEAKS^16^. For the statistical analysis, the dataset was median-normalized by normalyzer and a two-sided T-test was used to determine significance (p < 0.05) between 240 min and 0 h.

### Globupain activity with FRET substrates

Internally quenched fluorgenic substrates MCA-Trp-Thr-Ile-Nle-Gly-Pro-Asp-Ala-Lys(DNP)-OH and MCA-Trp-Thr-Ile-Arg-Gly-Pro-Asp-Ala-Lys(DNP)-OH were synthesized and purified to > 95% by GenScript (Supplementary File 1). Globupain was assayed at a concentration of 0.26 μg/mL with 50 μM of each substrate at 50 °C for 1 h in Assay Buffer 2. Fluorescence was measured at an excitation wavelength of 320 nm and an emission wavelength of 420 nm using a BioTek Synergy HTX Multimode Reader. All assays were performed in triplicate using a black 384-well plate.

### Effect of inhibitors on globupain activity

Increasing concentrations (7.45 nM to 20 μM) of commercially available protease inhibitors, Leupeptin (Research Products International) and MG101 (MedChemExpress), were preincubated with activated globupain (0.26 µg/mL) for 20 min at room temperature. Then, Boc-QAR-amc was added to a final concentration of 50 μM, and fluorescence was measured over 1 h at 50 °C in triplicate. The reaction velocity was calculated and plotted as a dose–response curve, normalized to a control with no inhibitor added. The dose–response curve was fitted using GraphPad Prism, version 10.4.1.

## Results

### Cleavage efficiency of globupain with protein substrates

**Fig. 1:**
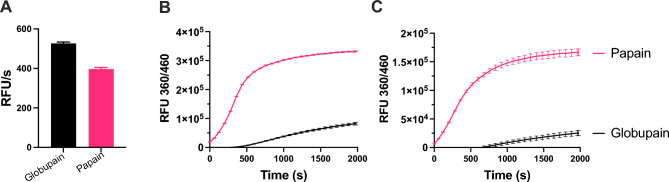
Comparing proteolytic activities of globupain and papain at 60 °C. (**A**) Activity of globupain against Boc-QAR-amc was compared to that of papain in Assay Buffer 1. Both enzymes were tested at a final concentration of 1.45 µg/mL. The reaction rates in relative fluorescence units per second (RFU/s) were calculated over 440 s. (**B**) Activity of globupain and papain measured over 2000s using fluorescently-labelled gelatin and collagen (**C**) as substrate. Error bars represent standard error of mean in all panels.

To compare the activity of globupain with that of the commercial cysteine protease, papain, we first identified assay buffer conditions in which both enzymes were functional and where globupain retained the same activity as previously reported^20^. Using the same concentration of each enzyme, we found that the tripeptide substrate Boc-QAR-amc was cleaved efficiently by both papain and globupain (Fig. 1A). These results confirm that papain can cleave peptides with Arg in the P1 position, even though arginine is not the optimal amino acid at this site^19^. We next compared the ability of globupain and papain to hydrolyze the protein substrates, gelatin, and collagen. Using fluorescently labelled gelatin, we observed that papain-mediated cleavage was detectable immediately upon enzyme addition and continued for approx. ~ 600 s before the rate of fluorescence change slowed. Under the same conditions, globupain-mediated cleavage was not detectable until after 300 s of incubation, after which fluorescence steadily increased until the end of the assay (2000s) (Fig. 1B). Similarly, using collagen as a substrate, papain hydrolyzed the protein efficiently within the first 600 s, whereas cleavage by globupain was not detectable until after 600 s (Fig. 1C). Together, these findings demonstrate that while both globupain and papain can cleave substrates with arginine at the P1 position at comparable rates, globupain hydrolyzes protein substrates, such as gelatin and collagen, at a much slower rate than papain. This difference is likely due to papain’s broader substrate specificity, allowing it to cleave at more sites within the substrate proteins. This observation prompted a more in-depth analysis of globupain’s activity and substrate specificity.

### Effects of salt, reducing reagent, and divalent cations on proteolytic activity

**Fig. 2:**
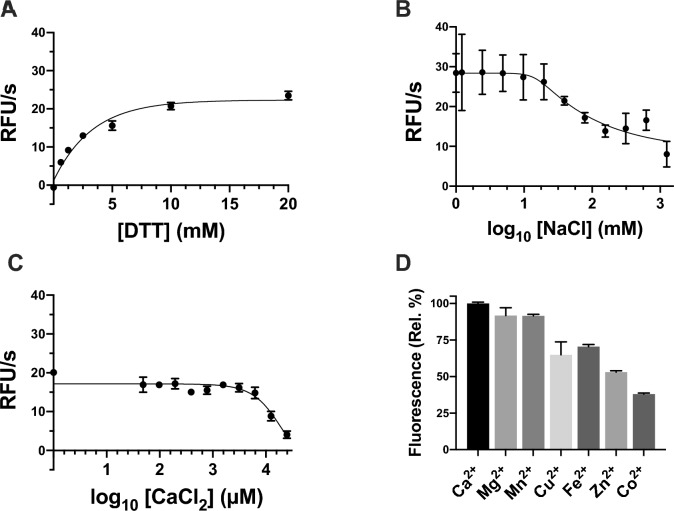
Effects of DTT (**A**), sodium chloride (**B**), calcium chloride (**C**), and common divalent cations (**D**), on globupain activity, assayed against the Boc-QAR-amc fluorescent substrate. Globupain activity was tested in the presence of increasing concentrations of DTT (**A**), NaCl (**B**) and CaCl_2_ (**C**). An increase in activity was observed with increasing DTT concentrations up to 20 mM, while maximum activity was retained in the presence of up to 1250 mM NaCl and 25 mM CaCl_2_. The effect of 1 mM of common divalent cations on globupain activity were also tested (**D**) and shown as percentage of fluorescence relative to the control ion Ca^2+^ (100%). Error bars represent standard error of mean in all panels.

Previously, globupain was assayed in a reaction buffer containing 150 mM NaCl, 10 mM DTT, and 1 mM CaCl_2_. However, it was unclear whether these conditions were optimal for enzyme activity. To address this, we assayed the enzyme under varying concentrations of DTT, NaCl, and CaCl_2_ while also testing different divalent cations for potential enhancement of activity. Enzyme activity increased sharply with DTT concentrations up to 5 mM but showed no further improvement at higher concentrations (Fig. 2A). These results suggest that the addition of DTT above 10 mM offers little benefit. Globupain was shown to be active across a wide range of NaCl concentrations; enzyme activity was highest at concentrations below 19 mM but declined progressively between 20 and 1250 mM NaCl (Fig. 2B). Notably, the standard assay buffer concentration of 150 mM NaCl was observed to reduce activity to 47% of the maximum. Regarding CaCl₂, enzyme activity was highest between 5 and 500 μM but declined at higher concentrations (Fig. 2C). Globupain retained 91.7%, 91.6%, and 65% of its mean activity in the presence of 1 mM of Mg^2+^, Mn^2+^, and Cu^2+^, respectively, compared to CaCl₂ (Fig. 2D). In contrast, activity decreased to 70.5%, 53%, and 38% when assayed with Fe^2+^, Zn^2+^, and Co^2+^, respectively.

### Effects of detergent on stability and catalytic activity

**Fig. 3:**
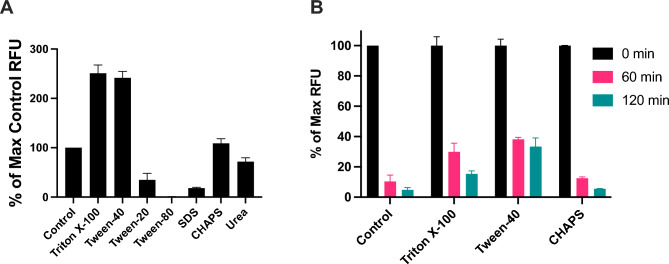
Effect of detergents and urea on globupain activity against the Boc-QAR-amc fluorescent substrate. The effects of 0.5% Triton X-100, Tween 20, Tween 40 and Tween 80 (non-ionic detergents), SDS (ionic detergent), CHAPS (zwitterionic detergent) and Urea (chaotropic agent) on globupain activity were tested (**A**). Values are displayed as percentage of the maximum control fluorescence, where the control column represents the activity of globupain without the addition of any detergents. A significant increase in activity is observed on samples with Triton X-100 and Tween 40 (**A**). Changes in the activity of globupain after pre-incubation with 0.5% each additive for 0, 60 and 120 min (**B**). Activity is represented as % of max fluorescence for each additive, which corresponds to the sample read at 0 min for each variant. Control column represents the activity of globupain without the addition of any detergents.

The susceptibility of globupain activity to various detergents, including zwitterionic (CHAPS), ionic (SDS), and non-ionic (Triton X-100, Tween 20, Tween 40, Tween 80) detergents, as well as the chaotropic agent urea was assessed at 75 °C against the Boc-QAR-amc substrate. At a final concentration of 0.5%, globupain activity increased in the presence of Triton X-100, Tween 40, and CHAPS, reaching 250.8%, 241.5%, and 108.8% of the control sample with no additives, respectively (Fig. 3A). Conversely, urea, Tween 20, and SDS reduced activity to 71.9%, 34.9%, and 18% of the control, respectively. Notably, Tween 80 completely abolished the proteolytic activity of globupain (Fig. 3A). Further analysis of the stimulatory effects of Triton X-100 and Tween 40 revealed that even at a concentration as low as 0.001%, these detergents significantly enhanced globupain’s catalytic activity (Fig. 3B,C).

The stability of globupain over time was assessed in the presence of Triton X-100, Tween 40, and CHAPS. When pre-incubated at 75 °C, globupain retained more of its activity in the presence of Triton X-100 and Tween 40 than without additives (Fig. 3B). Only 10.6% and 4.8% residual activity was observed after 60 and 120 min, respectively, compared to an enzyme sample maintained on ice for 120 min (Fig. 3B). These findings align with previous studies, which indicate that globupain is unstable at 75 °C and pH 7.1 due to autodigestion^20^.

Since detergents can affect enzyme stability, we evaluated the thermal stability of globupain across a temperature gradient in the presence of relevant detergents and urea using nano differential scanning fluorimetry. Intrinsic protein fluorescence was measured over the temperature range of 20 to 110 °C (Supplementary Fig. S1). In the absence of detergents, activated globupain had a melting temperature (T_m_) of 87.65 ± 0.20 °C. The addition of 1% SDS reduced thermal stability, lowering the T_m_ to 85.45 ± 0.18 °C (Table 1). In contrast, 1% Tween 20, Tween 40, and CHAPS increased the T_m_ of globupain to 92.14 ± 0.06 °C, 90.77 ± 0.25 °C and 92.00 ± 0.08 °C, respectively. For 1% urea (87.47 ± 0.03 °C) and Triton X-100 (87.53 ± 0.57 °C), no significant effect was observed. Due to its incompatibility with globupain, Tween 80 was excluded from this analysis.

**Table 1:** The effect of selected detergents on globupain thermal stability as determined by nanoDSF.

Sample	Tm [°C] ± SD
Activated globupain	87.65 ± 0.20
Activated globupain + 1% Tween 20	92.14 ± 0.06
Activated globupain + 1% Tween 40	90.77 ± 0.25
Activated globupain + 1% Triton X-100	87.53 ± 0.57
Activated globupain + 1% SDS	85.45 ± 0.18
Activated globupain + 1% urea	87.47 ± 0.03
Activated globupain + 1% CHAPS	92.00 ± 0.08

All experiments were carried out in triplicate; mean and standard deviation (SD) were calculated for all three measurements.

### Substrate profiling

**Fig. 4:**
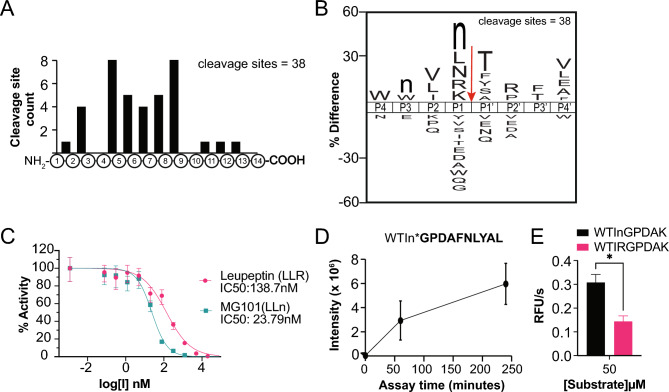
Multiplex substrate profiling by mass spectrometry (MSP-MS) was performed with globupain at pH 7.1. The sequences included in the data were found to be statistically significant (p < 0.05) and had a fold-change of 8 at the 4 h time point relative to the 0 h timepoint. All timepoints were taken in quadruplicates. (**A**) Observed globupain cleavage locations. Frequencies of cleavage locations within the 14-mer peptides were counted and graphed. The figure shows how many cleavages were found at each P1 location in the full 14 aa peptide. (**B**) IceLogo of globupain cleavage products. Sequences were imported into iceLogo software and compared against a negative dataset which includes all possible cleavage locations of peptides in the peptide mix. Enriched amino acids at each location are found above the X-axis, with the size of the letter corresponding to the percent enrichment. n, norleucine. Data is shown for P4, P3, P2, P1, P1ʹ, P2ʹ, P3ʹ, P4ʹ positions. (**C**) Dose response curves of two inhibitors, leupeptin and MG101, against globupain. The substrate Boc-QAR-amc was used. Activity was normalized to a vehicle treated sample. Data is the average ± SEM (n = 3). (**D**) Intensity of the cleavage product GPDAFNLYAL detected over time via mass spectrometry in the MSP-MS assay. (**E**) Comparison of cleavage rates of two substrates, WTInGPDAK and WTIRGPDAK, following incubation at 50 °C with globupain. Data is the average ± SEM (n = 3). A t-test was performed to assess significance (p < 0.05).

To further characterize the enzyme activity of globupain, multiplex substrate profiling by mass spectrometry (MSP-MS) was performed^16,17^. Globupain was incubated with a mixture of 228 peptides and incubated for up to 4 h. Out of the 2964 available peptide bonds with this peptide library, globupain cleaved at 38 sites where the cleavage product was significantly increased (eightfold change, q < 0.05) relative to the control assay containing an inactive enzyme. When the distribution of cleavage sites across the 14-mer peptides were assessed, globupain was found to preferentially cleave at sites between the 4th and 9th amino acids (Fig. 4A). These data indicate that globupain is an endopeptidase that has a preference for cleaving at a distance from the N- and C-termini. When the cleaved peptides were plotted as an iceLogo, a visual aid that shows enriched and de-enriched amino acids at each location surrounding a cleavage site, globupain was shown to prefer cleaving peptides that have norleucine (Nle), Leu, Asn, Arg and Lys, in the P1 position (Fig. 4B). The enrichment of Lys and Arg at P1 is consistent with other family C11 enzymes, which also favor these positively charged amino acids; however, a preference for Nle, Asn and Leu at P1 appears to be unique to globupain. The P2 position was found to prefer hydrophobic side chains, such as Val, Leu, and Ile. Lastly, the P1′ position prefers either the polar amino acids Thr or Ser, or the hydrophobic amino acids Phe, Tyr or Ala.

Since globupain can cleave substrates containing Nle in the P1 position, we predicted that it may also be inhibited by MG101, a commonly used calpain inhibitor consisting of acetyl-Leu-Leu-Nle-aldehyde. We assayed globupain with concentrations ranging from 1 pM to 80 µM of MG101 and generated a dose–response curve (Fig. 4C), calculating an IC_50_ of 23.79 nM ± 3.6 nM. We then compared the potency with a related inhibitor, leupeptin, which consists of acetyl-Leu-Leu-Arginine-aldehyde, and found the potency to be 5- to sixfold weaker (IC_50_ = 138.7 nM ± 25.6 nM). These results highlight the importance of the P1 Nle residue for binding to the S1 pocket of globupain.

Finally, to support the findings of the MSP-MS and inhibitor assay, we identified a peptide sequence in the library that was cleaved with the highest efficiency. This substrate contains the sequence, WTInGPDAFNLYAL and was cleaved between Nle (n) and Gly (G) (Fig. 4D). We synthesized a fluorogenic reporter substrate that contains only the P4 to P4′ amino acids of this sequence (WTInGPDA), flanked on the N-terminus by the fluorogenic reporter group, 7-amino-4-methylcoumarin and on the C-terminus by the quencher, 2,4-dinitrophenol. We showed that this substrate is cleaved by globupain over time (Fig. 4E). In addition, to compare Nle and Arg in the P1 position, we made a homologous substrate (WTIRGPDA) that differs only by the P1 residue. This substrate was also cleaved by globupain but with lower efficiency compared to WTInGPDA. These data confirm that substrates with P1-Nle are more efficiently cleaved than those with P1-Arg, providing us with a new substrate for future biochemical studies of globupain.

## Discussion

This study shows that the thermostable marine-derived clostripain-like cysteine protease, globupain^20^, is an endopeptidase with novel substrate specificity and tolerance to various chemical additives. Previously, we demonstrated that globupain favors Arg at the P1 position, with Boc-QAR-amc identified as a preferred substrate among the 17 fluorogenic compounds tested^20^. This finding is consistent with the characteristics of family C11 proteases, such as clostripain^21^ and PmC11 from the human gut bacterium^18^. However, when globupain activity was tested using Ac-VLTK-amc, the optimal fluorogenic substrate for PmC11, it was cleaved less efficiently than globupain’s preferred substrate, Boc-QAR-amc^20^. This result highlights differences in substrate cleavage preferences between the two proteases. To obtain more precise information about the substrate specificity of globupain and to compare this with that of PmC11^18^ and papain^19^, respectively,we therefore performed MSP-MS (Fig. 4A,B). This method enables the identification of a protease’s unique peptide fingerprint and characterizes the protease as either an exopeptidase or endopeptidase. Our first analysis confirmed that globupain preferentially cleaves at Arg and Lys in the P1 position. However, the comprehensive approach presented in this study revealed that globupain has a unique preference for Nle (employed in MSP-MS as a substitute for Met), Asn and Leu at the P1 position, which distinguishes globupain from PmC11^18^, clostripain or other C11 family proteases cataloged in the MEROPS database^1^.

Globupain carries the conserved catalytic His/Cys dyad of cysteine peptidases despite bearing a low sequence similarity of 24.2% to PmC11 and 23.5% to clostripain, respectively^20^. Interestingly, despite any notable sequence similarity between them, a preference for Nle and Lys in the P1 position was previously also observed for papain, a protease with a wide range of commercial applications across various industries^22^. Where globupain prefers Nle > Leu > Asn > Arg > Lys in the P1 position, papain prefers Lys > Nle > Gly. Unlike the more selective clostripain-like proteases of the C11 family, papain belongs to the C1 family in the MEROPS database^1^. Papain exhibits broad substrate specificity, where the rate of cleavage is primarily driven by the amino acids in the P2 position, favoring short aliphatic (Val, Ile, Leu, Ala) and polar (Ser, Thr) amino acids^19^. In contrast, globupain shows a narrower preference in the P2 position, favoring Val, Ile, and Leu, respectively. The more restricted amino acid preference of globupain compared to papain was evident when their hydrolytic activity was assessed using complex proteins, such as gelatin and collagen, as substrates, where papain was significantly more efficient than globupain (Fig. 1B).

Finding targeted inhibitors that can stop uncontrolled protease activity may be of value in applications involving proteases. For example, for research applications such as mass spectrometry sample preparation, protein processing or synthetic biology approaches, it may be desirable to inactivate the protease quickly. The MSP-MS-based peptide substrate fingerprinting approach offers a streamlined and comprehensive method for identifying effective inhibitors of the protease under study. For globupain, we found that the two commercially available calpain inhibitors, MG101 and leupeptin, were both highly effective at nanomolar concentrations (Fig. 4C). Consistent with the substrate specificity profile of globupain (Fig. 4B), MG101, a tripeptide inhibitor with the sequence LLn, showed a stronger inhibitory effect than Leupeptin, which has the sequence LLR. Building on our previous knowledge of the activation mechanism of globupain^20^, we now have a deeper understanding of how to apply and control its proteolytic activity.

We have previously demonstrated that globupain is both thermoactive and thermotolerant^20^. Here, we assessed the effects of selected detergents and urea added at a concentration of 1% and found that globupain remained thermotolerant under all tested conditions, with only minor changes in stability (Table 1). However, the catalytic activity of globupain was modulated differently by the tested compounds. While Triton X-100 and Tween 40 enhanced both activity and stability, the addition of SDS, Tween 80, Tween 20, and urea negatively affected enzyme activity (Fig. 3A,B). It is worth noting that all detergents were used at concentrations higher than their critical micelle concentrations (CMCs). For Triton X-100, which significantly influenced globupain activity, micelles are formed at a CMC of 0.016% (0.26 mM), and their size has been shown to increase at temperatures above 40 °C^23^. The higher micellar growth is also induced by elevated salt concentration. This effect is attributed to the salt’s ability to sequester water molecules, leading to micelle swelling^24^. It is widely recognized that observed enhanced enzymatic activity arises from the binding of detergent molecules to the protein surface through both hydrophobic and polar interactions^25^. These interactions can stabilize the enzyme’s tertiary structure by creating a microenvironment that mimics cytosolic conditions. However, detergents may also induce conformational changes in proteins, potentially affecting their enzymatic activity and thermodynamic properties^26–28^. Recently, a highly stimulatory effect was observed for a caseinolytic protease from *Cobetia amphilecti* when its activity was measured in a reaction mixture supplemented with 1% Triton X-100^29^. The complete inhibition of glubupain activity by Tween 80 contrasts with what has been reported for other proteases, such as serine alkaline protease from *Bacillus cereus*^30^. For this protease, enzymatic activity increased by 25% with the addition of 1% Tween 80, whereas a 15% decrease in activity was observed with Triton X-100. For the commercially available serine alkaline protease, Alcalase® from *Bacillus licheniformis*, the addition of 10% Tween 80 did not significantly affect enzyme activity^31^. However, the addition of 10% Tween 40 or Triton X-100 increased the enzyme activity by 25% and 35%, respectively. Therefore, globupain may be attractive in detergent formulations where Tween 40 or Triton X-100 is used. For detergent formulations requiring the presence of Tween 80, however, globupain is observed to be incompatible.

Globupain also showed distinctive properties when tested alongside other additives known to affect the activities of similar proteases, such as the common reducing agent DTT^32^, NaCl and CaCl₂, and various relevant divalent cations^33^. The enzyme demonstrated a high tolerance for DTT and remained fully active even at concentrations up to 20 mM (Fig. 2A). Notably, while DTT is known to inhibit collagenase from *Clostridium histolyticum*, it does not impede clostripain at a concentration of 10 mM^34^. Additionally, globupain showed tolerance to NaCl and CaCl₂; however, higher concentrations of both salts resulted in a reduction in its activity (Fig. 2B,C). Altogether, globupain could remain active in applications that operate under highly reducing conditions and across a wide range of salt concentrations.

Divalent metal cations added in the millimolar range are known to affect the proteolytic activity of various proteases from different families, including clostripains^1,35^. In this study, we observed that substituting Ca^2+^ with Mg^2+^ and Mn^2+^ at 1 mM concentrations resulted in a minor reduction of mean activity to 91.71% and 91.58%, respectively (Fig. 2D). This finding aligns with previous observations of PmC11 retaining its activity in the presence of Mn^2+^ and Mg^2+^^36^. However, whereas the addition of Cu^2+^ at a concentration of 1 mM completely inhibited PmC11 activity, globupain maintained 65% of its activity compared to the addition of Ca^2+^. The most inhibitory divalent cation for globupain was found to be Co^2+^, which resulted in a remaining activity of 37.74%. This is in strong contrast to PmC11, where 1 mM Co^2+^ did not appear to inhibit enzyme activity at all^36^.

In conclusion, we present an innovative and efficient approach for characterizing proteases with potential biotechnological applications by determining their substrate preferences using MSP-MS, which can inform the identification of efficient inhibitors for the respective protease. Altogether, our approach opens new avenues for the discovery of novel enzymatic properties of marine proteases and their potential application.

## Supplementary Information

Supplementary Figure S1.

Supplementary Information.

## Data Availability

The native C11 globupain protease is available in GenBank under the accession number OQ718499. Mass spectrometry data is publicly available on UCSD’s Mass Spectrometry Interactive Virtual Environment (MassIVE): MSV000097108, and ProteomeXchange: PXD060732.
