# Supplementary material for: Substrate profiling of marine-derived thermotolerant cysteine protease reveals unique cleavage preferences for industrial applications
Source: Sci Rep. 2025 Jul 21;15:26481. doi: 10.1038/s41598-025-11635-1 (PMC12280095; doi:10.1038/s41598-025-11635-1)
Supplement: Supplementary file 1 — Supplementary Figure S1. [file 41598_2025_11635_MOESM1_ESM.pdf]

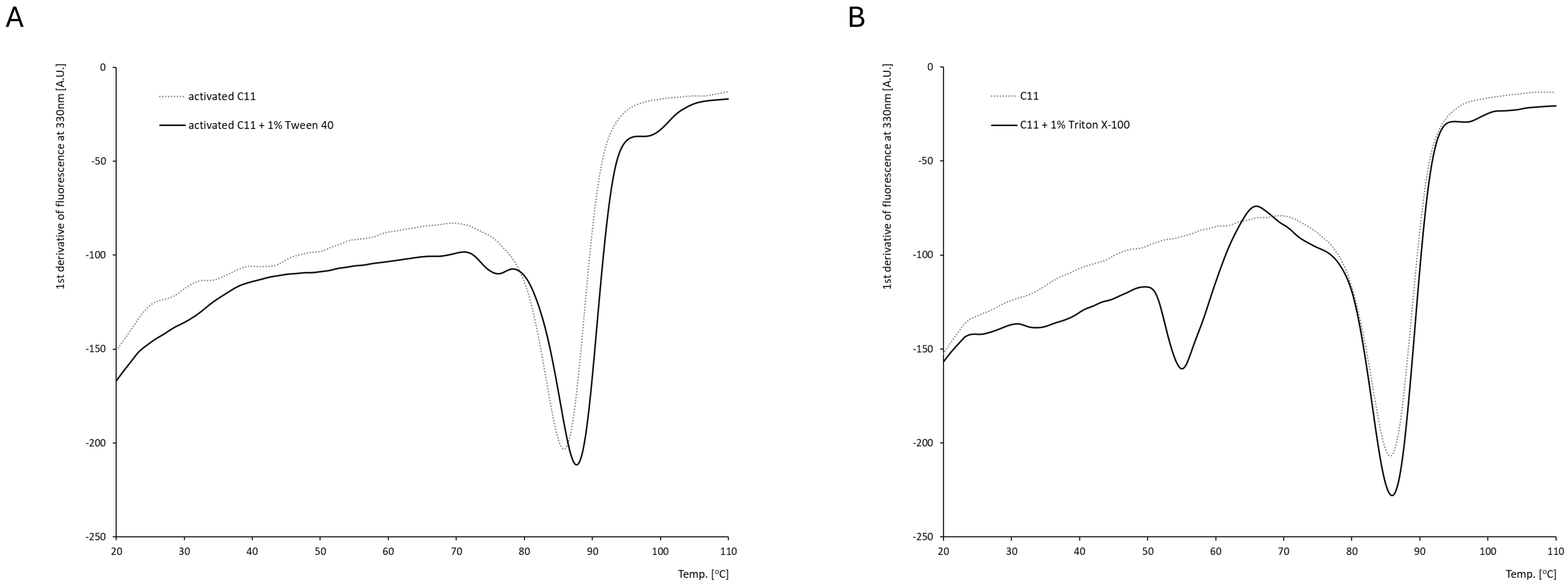


**Supplementary Figure S1:** The effect of 1% Tween 40 and Triton X-100 (non-ionic detergents) on the thermal stability of globupain C11. Unfolding profiles of globupain in the presence of 1% Tween 40 (A) and 1% Triton X-100 (B) were analyzed using nanoDSF. The dotted line represents the melting curve of activated globupain C11 alone, while the solid line shows the melting curves of globupain in the presence of the detergents.
