# Supplementary material for: Substrate profiling of marine-derived thermotolerant cysteine protease reveals unique cleavage preferences for industrial applications
Source: Sci Rep. 2025 Jul 21;15:26481. doi: 10.1038/s41598-025-11635-1 (PMC12280095; doi:10.1038/s41598-025-11635-1)
Supplement: Supplementary file 2 — Supplementary Information. [file 41598_2025_11635_MOESM2_ESM.pdf]

Supplementary File 1: Custom fluorogenic peptides were synthesized by Genscript and the synthesis was confirmed via mass spectrometry.

Sample Name :WTInGPDA
Sample ID :U7604456G0-1
Time Processed :10:22:44
Month-Day-Year Processed :10/04/2023

Pump A : 0.065% trifluoroacetic in 100% water (v/v)
Pump B : 0.05% trifluoroacetic in 100% acetonitrile (v/v)
Total Flow:1 ml/min
Wavelength:220 nm
<<LC Time Program>>

Time	Module	Command	Value
0.01	Pumps	B.Conc	5
25.00	Pumps	B.Conc	65
25.01	Pumps	B.Conc	95
27.00	Pumps	B.Conc	95
27.01	Pumps	B.Conc	5
35.00	Pumps	B.Conc	5
35.01	Controller	Stop	

<<Column Performance>>
<Detector A>
Column :Inertsil ODS-SP 4.6 x 250 mm
Equipment: GR11010440

<Chromatogram>

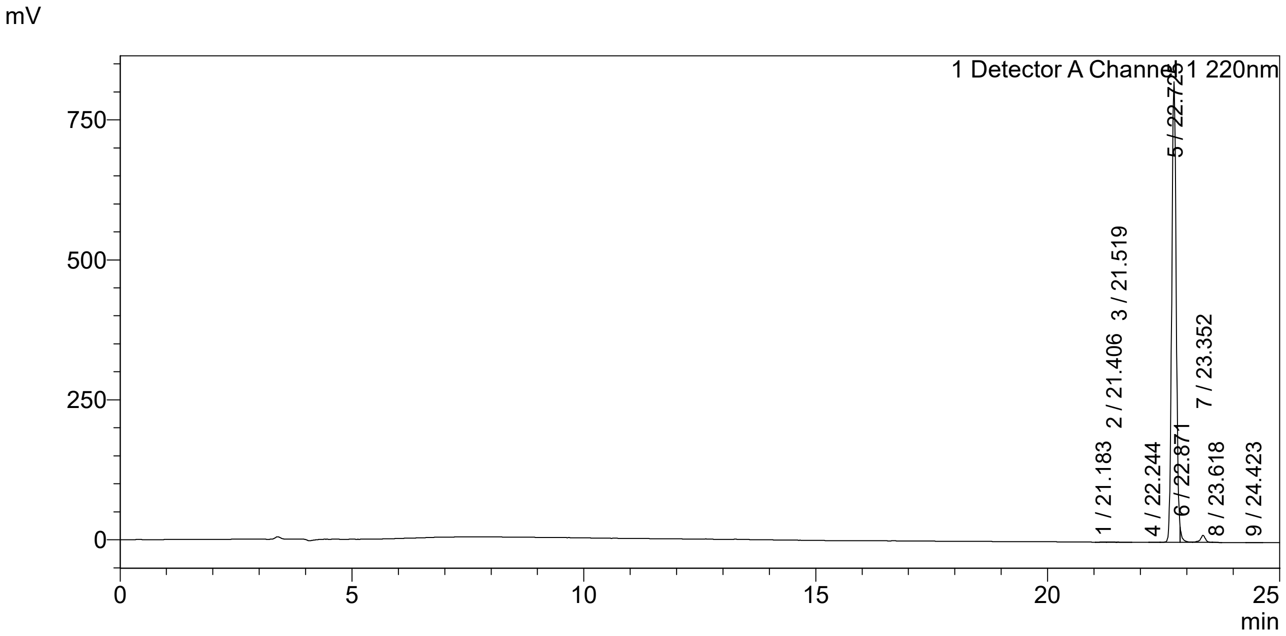

<Peak Table>

Detector A Channel 1 220nm

Peak#	Ret. Time	Area	Height	Area%
1	21.183	1836	186	0.032
2	21.406	2611	240	0.046
3	21.519	2086	189	0.037
4	22.244	1498	143	0.026
5	22.725	5491483	822879	96.997
6	22.871	64523	22035	1.140
7	23.352	93814	12421	1.657
8	23.618	1919	329	0.034
9	24.423	1725	140	0.030
Total		5661497	858562	100.000

Mass Spectrum

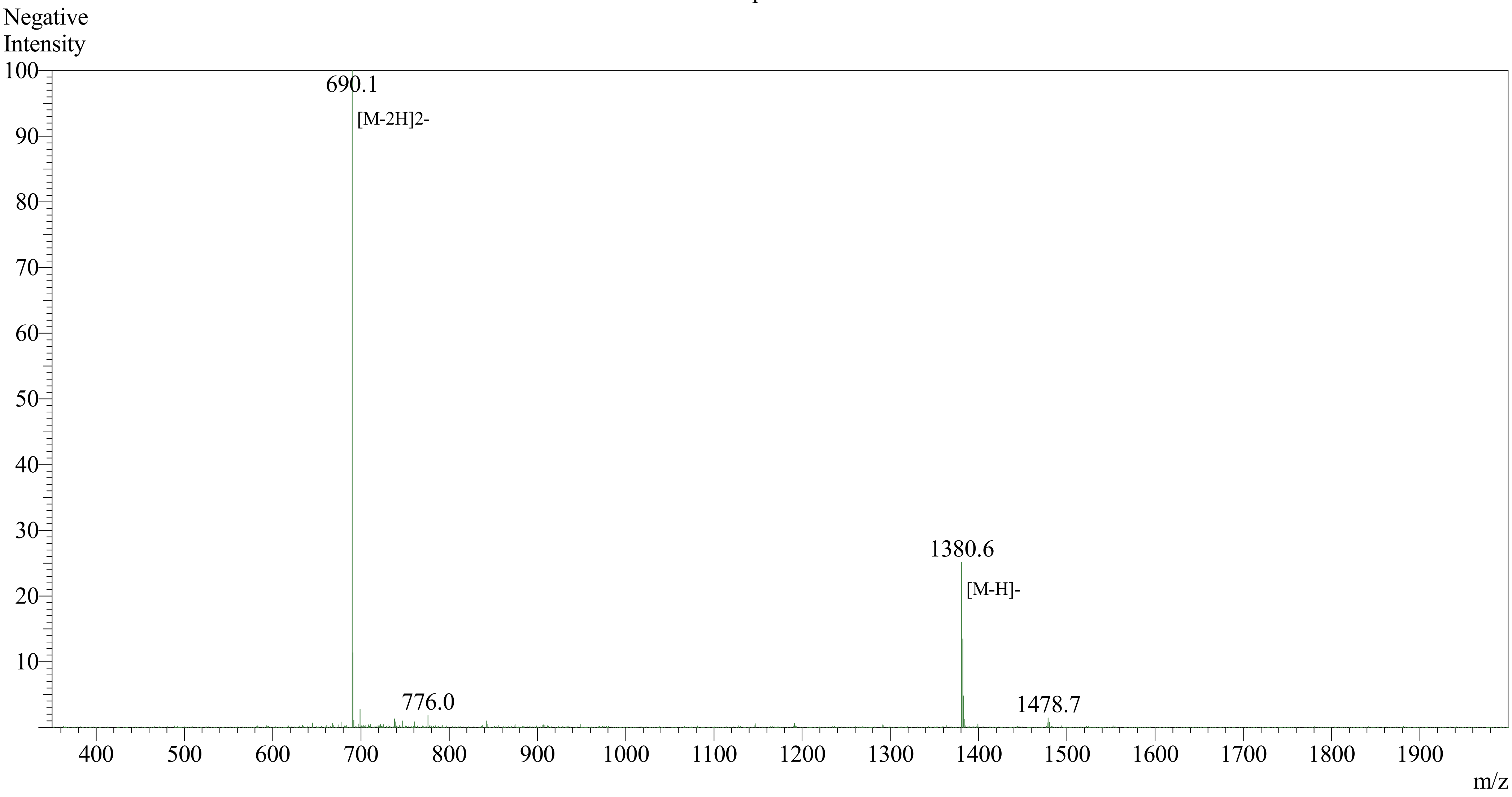

Sample Information
Month-Day Processed : 09/27/23
Time Processed : 11:48:39
Injection Volume : 0.3
Sample Name : WTInGPDA
Sample ID : U7604456G0-1
Theoretical MW : 1382.46
Observed MW : 1382.2

Interface :ESI
Nebulizing Gas Flow :1.5L/min
CDL Temp :250
Block Temp :200

Equipment : ZJ21010035
Interface Bias : -3.5 kV
Drying Gas Flow :5 L/min
T.Flow :0.2 ml/min
B.conc :50%H2O/50%MeOH

Sample Name :WTIRGPDA
Sample ID :U7604456G0-3
Time Processed :23:47:42
Month-Day-Year Processed :09/02/2023

Pump A : 0.065% trifluoroacetic in 100% water (v/v)
Pump B : 0.05% trifluoroacetic in 100% acetonitrile (v/v)
Total Flow:1 ml/min
Wavelength:220 nm
<<LC Time Program>>

Time	Module	Command	Value
0.01	Pumps	B.Conc	5
25.00	Pumps	B.Conc	65
25.01	Pumps	B.Conc	95
27.00	Pumps	B.Conc	95
27.01	Pumps	B.Conc	5
35.00	Pumps	B.Conc	5
35.01	Controller	Stop	

<<Column Performance>>
<Detector A>
Column :Inertsil ODS-SP 4.6 x 250 mm
Equipment: GK12010012

<Chromatogram>

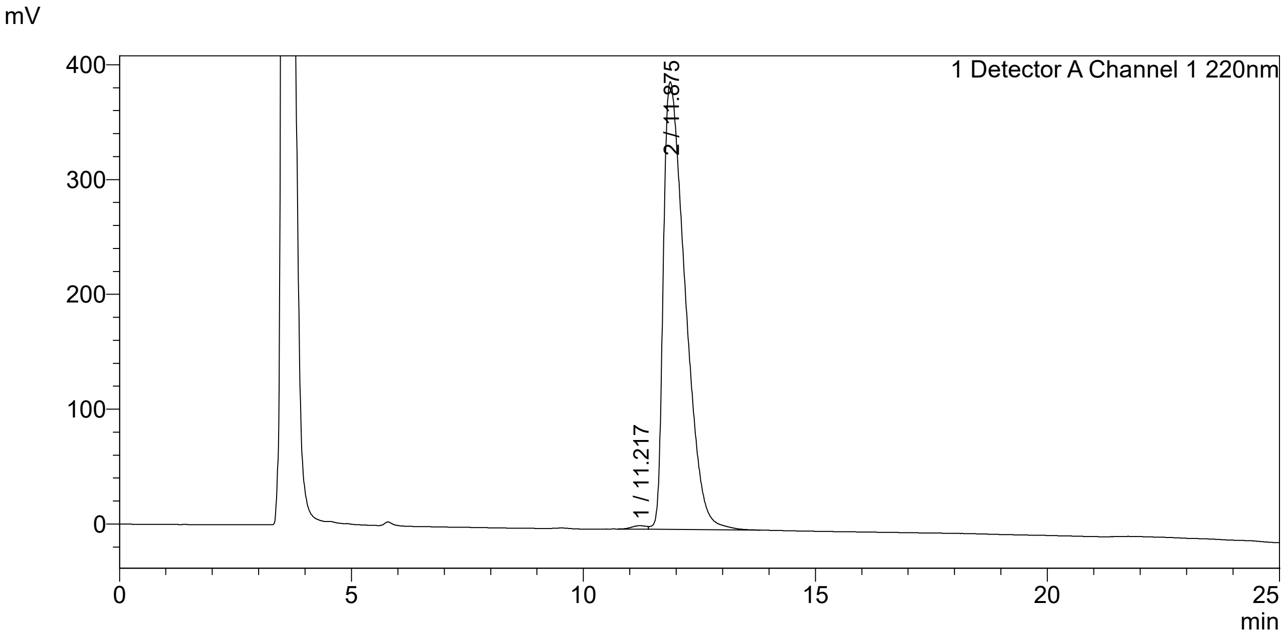

<Peak Table>

Detector A Channel 1 220nm

Peak#	Ret. Time	Area	Height	Area%
1	11.217	67228	3062	0.524
2	11.875	12751244	390043	99.476
Total		12818471	393105	100.000

Mass Spectrum

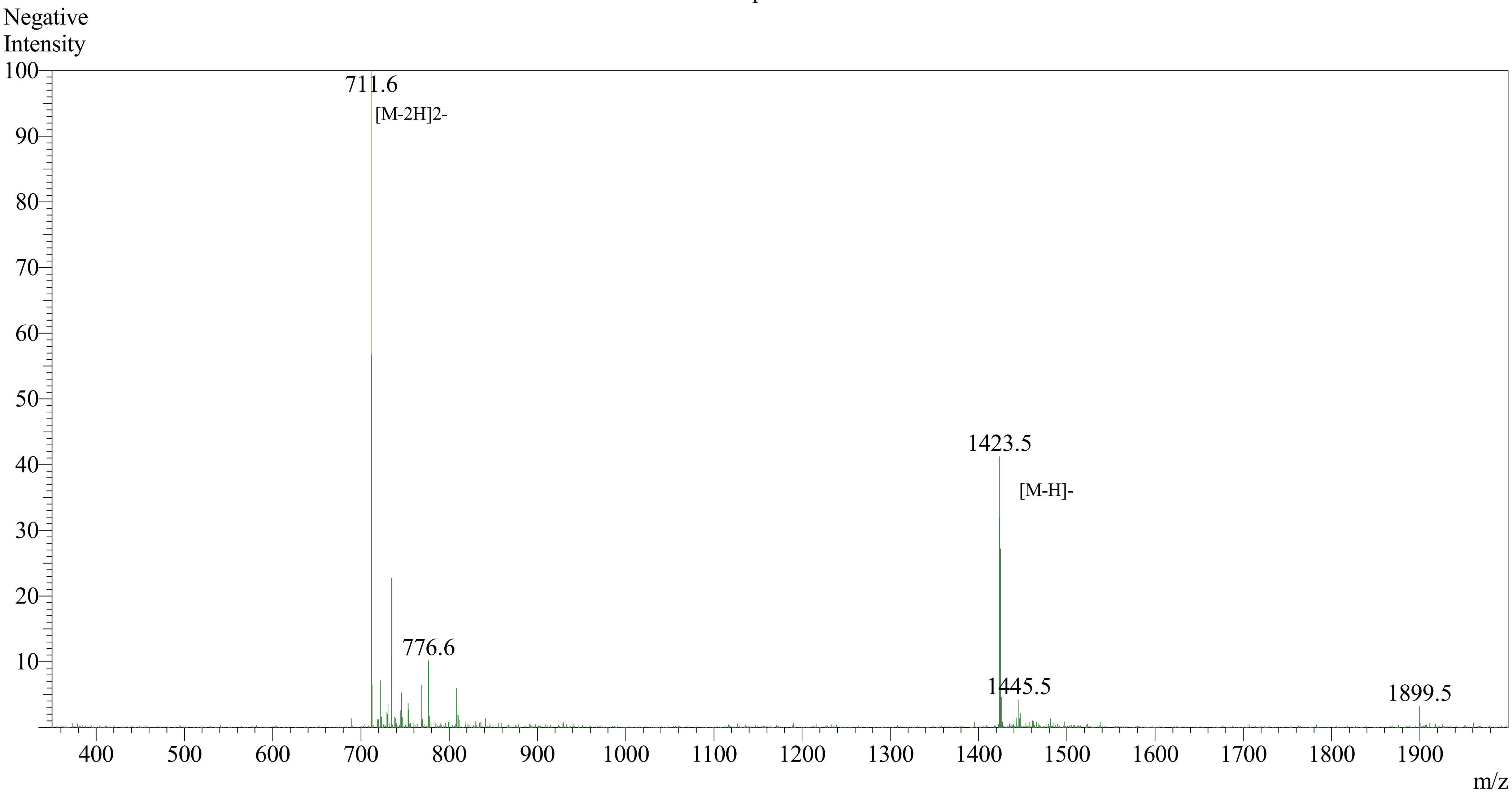

Sample Information
Month-Day Processed : 09/03/23
Time Processed : 8:14:56
Injection Volume : 0.3
Sample Name : WTIRGPDA
Sample ID : U7604456G0-3
Theoretical MW : 1425.49
Observed MW : 1425.2

Interface :ESI
Nebulizing Gas Flow :1.5L/min
CDL Temp :250
Block Temp :200

Equipment : ZJ21010035
Interface Bias : -3.5 kV
Drying Gas Flow :5 L/min
T.Flow :0.2 ml/min
B.conc :50%H2O/50%MeOH
